# Lower-Limb Biomechanical Adaptations to Exercise-Induced Fatigue During Running: A Systematic Review of Injury-Relevant Mechanical Changes

**DOI:** 10.3390/life16020272

**Published:** 2026-02-04

**Authors:** Prashant Kumar Choudhary, Suchishrava Choudhary, Sohom Saha, Yajuvendra Singh Rajpoot, Vasile-Cătălin Ciocan, Voinea Nicolae-Lucian, Silviu-Ioan Pavel, Constantin Șufaru

**Affiliations:** 1 Department of Physical Education Pedagogy, Lakshmibai National Institute of Physical Education, Gwalior 474002, Madhya Pradesh, India; prashantlnipe2014@gmail.com (P.K.C.); suchishrava05@gmail.com (S.C.); 2 Department of Sport Psychology, Lakshmibai National Institute of Physical Education, Gwalior 474002, Madhya Pradesh, India; sohomsaha77@gmail.com; 3 Department of Sports Management & Coaching, Lakshmibai National Institute of Physical Education, Gwalior 474002, Madhya Pradesh, India; yajupitu25@gmail.com; 4 Faculty of Movement, Sports, and Health Sciences, “Vasile Alecsandri” University of Bacău, 600115 Bacău, Romania; silviu.pavel@ub.ro (S.-I.P.); sufaruconstantin@ub.ro (C.Ș.)

**Keywords:** running biomechanics, exercise-induced fatigue, lower-limb mechanics, injury risk, spring-mass behaviour, impact loading

## Abstract

**Background/Objectives:** Exercise-induced fatigue is a fundamental component of running performance and training, yet it is also implicated in altered movement mechanics and increased injury risk. While numerous studies have examined fatigue-related biomechanical changes during running, findings remain fragmented across biomechanical domains and fatigue modalities. The purpose of this systematic review was to synthesize contemporary evidence on the effects of fatigue on lower-limb biomechanics during running and to interpret the potential injury relevance of these adaptations. **Methods:** A systematic literature search was conducted in PubMed, Scopus, and Web of Science for original empirical studies published between January 2010 and December 2025. Eligible studies involved human participants performing running or running-related tasks, applied an explicit fatigue protocol, and reported quantitative lower-limb biomechanical outcomes. Study selection followed PRISMA 2020 guidelines. Data extraction included participant characteristics, fatigue protocols, biomechanical measures, instrumentation, and key findings. Methodological quality was assessed using the Cochrane Risk of Bias 2 (RoB-2) tool. Due to substantial methodological heterogeneity, findings were synthesized narratively. **Results:** Twenty-four studies met the inclusion criteria. Across studies, fatigue consistently altered spatiotemporal parameters, joint kinematic and kinetic variables, spring-mass behavior, impact loading, coordination variability, neuromuscular output, and inter-limb symmetry. Common adaptations included increased ground contact time, reduced ankle joint power and stiffness, increased joint range of motion, elevated impact loading, and greater movement variability. These changes reflected reduced mechanical efficiency and a redistribution of mechanical load from distal to proximal joints, particularly toward the knee and hip. Similar fatigue-related biomechanical patterns were observed in both laboratory-based and real-world endurance running conditions. **Conclusions:** Exercise-induced fatigue produces systematic and injury-relevant alterations in lower-limb biomechanics during running. These adaptations may preserve short-term performance but create mechanical conditions associated with increased susceptibility to overuse and non-contact injuries. Integrating fatigue-aware biomechanical assessment, neuromuscular conditioning, and individualized load management strategies may help mitigate adverse fatigue-related adaptations.

## 1. Introduction

Regular involvement in sport and physical activity promotes adaptations in physical capacity and neuromuscular control, while also contributing positively to mental health and holistic development [1,2]. Despite its simplicity, running imposes substantial repetitive mechanical loads on the lower extremities, requiring efficient neuromuscular coordination, elastic energy storage, and precise joint control to maintain performance and minimize injury risk [3,4]. Fatigue, an inevitable consequence of prolonged or intense running, has been recognized as a critical factor influencing movement mechanics and injury susceptibility. Understanding how fatigue alters lower-limb biomechanics is therefore essential for advancing injury prevention strategies, optimizing training design, and improving performance sustainability.

Fatigue is a multifactorial phenomenon encompassing neuromuscular, metabolic, and central components, all of which can influence movement execution. In the context of running, fatigue has been shown to affect force production, coordination, and motor control, leading to altered movement strategies aimed at maintaining task performance [5]. These compensatory adaptations, while potentially beneficial in the short term, may simultaneously increase mechanical stress on musculoskeletal structures. Consequently, fatigue has been implicated as a contributing factor in both overuse injuries, such as stress fractures and tendinopathies, and acute non-contact injuries [6]. Biomechanical research over the past two decades has increasingly focused on identifying fatigue-induced changes in running mechanics. Experimental studies have reported fatigue-related alterations in spatiotemporal parameters, including prolonged ground contact time and modified stride characteristics, suggesting reduced neuromuscular efficiency and altered force application strategies [7,8]. Joint-level analyses further indicate that fatigue affects kinematics and kinetics across the ankle, knee, and hip, often resulting in reduced ankle power and stiffness and increased reliance on proximal joints to sustain locomotion [9,10]. Such proximal load redistribution has important implications for injury risk, particularly at the knee and hip. Spring-mass behaviour has emerged as a key conceptual framework for understanding fatigue-related mechanical adaptations during running. Several studies have demonstrated reductions in leg and vertical stiffness under fatigued conditions, reflecting diminished elastic energy storage and return [11,12]. Reduced stiffness may compromise shock attenuation capacity, thereby increasing impact transmission to passive tissues such as bone and cartilage. Indeed, fatigue-induced increases in impact loading variables, including vertical loading rate and tibial acceleration, have been reported in both laboratory-based protocols and real-world endurance events [13,14]. In addition to joint mechanics and impact loading, fatigue has been shown to influence coordination variability and inter-limb symmetry. Increased coordination variability at the trunk–pelvis–hip complex and altered motor variability structure have been observed following fatigue, particularly in novice runners [15,16]. While movement variability is a normal feature of adaptive motor control, excessive or poorly organized variability under fatigue may reflect compromised neuromuscular regulation and reduced movement stability. Similarly, fatigue-related increases in inter-limb asymmetry have been reported, indicating uneven load distribution that may predispose runners to unilateral injury development [17].

Despite extensive research on fatigue-related biomechanical adaptations in running, substantial heterogeneity exists in fatigue protocols, participant characteristics, running environments, and biomechanical outcome measures. Fatigue has been induced using diverse modalities, from short-duration sprint tasks to prolonged endurance efforts—eliciting distinct physiological and mechanical responses that limit statistical comparability. Consequently, a narrative synthesis was adopted to integrate findings across biomechanical domains while preserving contextual and mechanistic interpretation where meta-analysis is inappropriate.

Nevertheless, the growing body of research on fatigue-related running biomechanics remains fragmented. Studies vary widely in fatigue protocols, participant characteristics, running environments, biomechanical outcome measures, and verification of fatigue. Moreover, while individual studies provide valuable insights, no consensus has yet emerged regarding the consistency, direction, and injury relevance of fatigue-induced biomechanical changes across different contexts. Importantly, systematic reviews that focus exclusively on synthesizing original experimental evidence while deliberately excluding secondary analyses such as meta-analyses and bibliometric studies remain relatively limited in the running biomechanics literature.

Exercise-induced fatigue is a multifactorial construct encompassing interacting central, peripheral, neuromuscular, and metabolic mechanisms. Central fatigue reflects reductions in neural drive originating from the central nervous system, whereas peripheral fatigue involves impairments in excitation-contraction coupling, muscle contractile capacity, and local energy availability. Metabolic fatigue is characterized by the accumulation of metabolites such as hydrogen ions, inorganic phosphate, and lactate, as well as substrate depletion during prolonged exercise, while neuromuscular fatigue reflects altered motor unit recruitment, synchronization, and force–time characteristics. The included studies targeted these mechanisms to varying degrees depending on fatigue modality: sprint- and task-based protocols predominantly elicited metabolic and neuromuscular fatigue; prolonged endurance protocols emphasized metabolic depletion and muscle damage; and repeated or high-intensity protocols incorporated combined central and peripheral contributions. This framework provides a physiological context for interpreting the heterogeneous biomechanical adaptations observed under fatigue.

Therefore, the purpose of this systematic review was to synthesize original empirical studies published between 2010 and 2025 that examined the effects of fatigue on lower-limb biomechanics during running. Specifically, this review aimed to (i) identify consistent fatigue-induced biomechanical adaptations across spatiotemporal, kinematic, kinetic, stiffness, impact, and coordination domains; (ii) evaluate the consistency of these adaptations across study designs and fatigue modalities; and (iii) interpret their potential relevance to injury-related mechanical loading. By providing an integrated synthesis of contemporary evidence, this review seeks to enhance understanding of fatigue-related biomechanical mechanisms and inform future research, training, and injury prevention strategies. Accordingly, the research question of this systematic review was framed using the PICO framework, where the population comprised, human participants engaged in running or running-related tasks across varying ages and training levels. The exposure of interest was exercise-induced fatigue, examined through running-, sprint-, endurance-, or task-based fatigue protocols, with comparisons made against non-fatigued or pre-fatigue conditions. Outcomes focused on quantitative lower-limb biomechanical measures, including spatiotemporal parameters, joint kinematics and kinetics, stiffness, impact loading, coordination variability, inter-limb asymmetry, and neuromuscular mechanical indicators.

## 2. Materials and Methods

### 2.1. Study Selection Procedures

**Figure 1:**
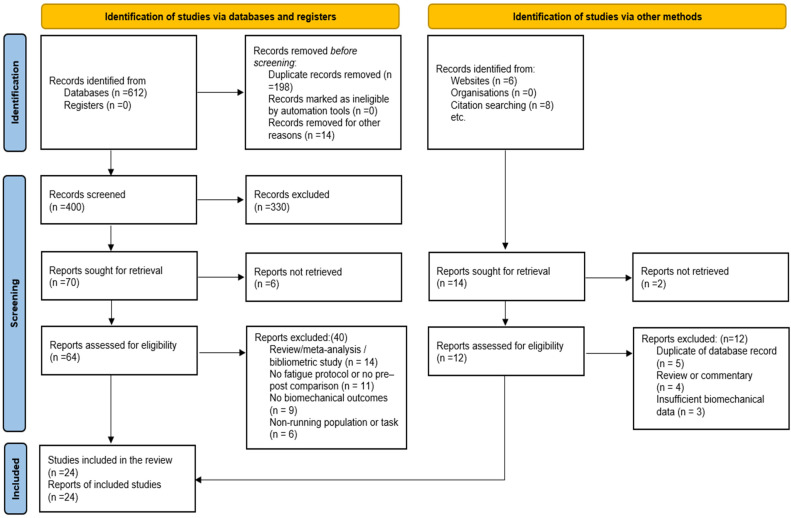
PRISMA 2020 flow diagram of the study selection process.

All records retrieved from the database search were imported into a reference management software, and duplicate records were removed before screening. Study selection was conducted in two stages. First, titles and abstracts were independently screened to exclude clearly irrelevant studies, such as those not involving running, not involving fatigue, or not reporting biomechanical outcomes. Second, full-text articles of potentially eligible studies were assessed against predefined inclusion and exclusion criteria. Studies were included if they involved human participants performing running or running-related tasks, employed an explicit fatigue protocol, and reported quantitative biomechanical outcomes of the lower limb. Systematic reviews, meta-analyses, bibliometric studies, non-running studies, and studies without pre-post fatigue biomechanical comparisons were excluded. Discrepancies during study selection were resolved through discussion, consistent with established systematic review methodology [18]. The complete selection process is summarized using a PRISMA 2020 flow diagram (Figure 1).

All included studies involved healthy participants without diagnosed neurological disorders or acute musculoskeletal injuries; no studies explicitly examined clinical populations or runners with active injury, which limits generalizability to rehabilitation or pathological cohorts.

### 2.2. Literature Search: Administration and Update

A systematic literature search was conducted to identify studies examining the effects of fatigue on lower-limb biomechanics during running. The search strategy was developed and reported in accordance with the PRISMA 2020 guidelines [19] and followed established recommendations for transparent reporting of electronic search strategies in systematic reviews [20]. Searches were implemented across three electronic databases, PubMed, Scopus, and Web of Science, selected for their comprehensive coverage of biomechanics, sports science, and kinesiology research. Search terms were constructed using combinations of keywords and Boolean operators related to running (“running,” “treadmill,” “overground running”), fatigue (“fatigue,” “exercise-induced fatigue,” “running-induced fatigue”), and biomechanics (“biomechanics,” “kinematics,” “kinetics,” “stiffness,” “impact loading,” “ground reaction forces”). The complete Boolean search strategy used in PubMed was as follows: (“running” OR “distance running” OR “treadmill running” OR “overground running” OR “sprint running”) AND (“fatigue” OR “exercise-induced fatigue” OR “running-induced fatigue” OR “neuromuscular fatigue”) AND (“biomechanics” OR “kinematics” OR “kinetics” OR “joint mechanics” OR “stiffness” OR “ground reaction force” OR “impact loading” OR “spring-mass” OR “movement variability”).

The search was limited to studies published in English between January 2010 and December 2025 to capture contemporary biomechanical methodologies. Reference lists of eligible studies and relevant reviews were manually screened to identify additional studies not retrieved through database searching. The final search update was performed before manuscript submission to ensure inclusion of the most recent evidence (Table 1).

### 2.3. Data Extraction

Data extraction was performed using a standardized extraction form designed specifically for biomechanics research. Extracted information included author and year of publication, study design, participant characteristics (sample size, sex, training status), fatigue protocol characteristics, biomechanical outcome domains, measurement instruments, and key findings related to fatigue-induced biomechanical changes. Particular emphasis was placed on extracting details of fatigue exposure and biomechanical measurement techniques to allow methodological comparison across studies. When required information was unclear or incomplete, the original article was carefully re-examined to minimize extraction errors. This approach follows best practice recommendations for systematic reviews in movement science and biomechanics [18,21,22].

### 2.4. Methodological Quality of the Included Studies

The methodological quality and risk of bias of the included studies were assessed using the Cochrane Risk of Bias 2 (RoB-2) tool, adapted for experimental biomechanics and kinesiology research [23]. Although the RoB-2 tool was originally developed for randomized controlled trials, it was pragmatically adapted in this review to evaluate within-subject experimental designs, with emphasis on measurement validity, protocol standardization, and selective reporting. The following domains were evaluated: bias arising from the randomization process, deviations from intended interventions (fatigue protocol adherence), missing outcome data, measurement of outcomes, and selective reporting. As most included studies employed non-randomized or within-subject experimental designs, particular attention was given to protocol standardization and outcome measurement validity. Each study was categorized as having low risk of bias, some concerns, or high risk of bias.

The RoB-2 tool was adapted because its domain-based framework aligns well with controlled experimental and within-subject biomechanics designs, where protocol standardization and objective outcome measurement are central. Although ROBINS-I was considered, it is primarily suited for observational clinical studies with complex confounding structures and was therefore less appropriate for tightly controlled laboratory fatigue experiments.

This assessment informed the interpretation of findings but did not serve as an exclusion criterion, in line with PRISMA recommendations.

Although the RoB-2 tool was originally developed for randomized controlled trials, it was applied in the present review with contextual adaptation due to the predominance of experimental, within-subject, and repeated-measures designs in biomechanics research. Several RoB-2 domains, particularly outcome measurement, missing data, and selective reporting, are directly applicable to fatigue biomechanics studies regardless of randomization. The randomization domain was interpreted with caution, acknowledging that many included studies employed controlled laboratory fatigue protocols rather than allocation-based group comparisons. Alternative tools designed for non-randomized intervention studies (e.g., ROBINS-I) were considered; however, they were deemed less appropriate given the acute, mechanistic nature of the fatigue protocols and the absence of exposure-based group comparisons. This pragmatic approach aligns with prior systematic reviews in sports biomechanics that adapt RoB-2 for experimental and within-subject designs to ensure consistent and transparent methodological appraisal.

### 2.5. Summary Measures

Due to heterogeneity in fatigue protocols, biomechanical outcome measures, and reporting metrics, standardized quantitative summary measures such as pooled effect sizes were not calculated. However, synthesis extended beyond simple vote counting. Where available, the magnitude and direction of biomechanical changes were qualitatively integrated into the narrative synthesis, including reported percentage changes, relative shifts in joint contribution, changes in stiffness magnitude, and alterations in force time characteristics. This approach emphasizes the mechanical significance of fatigue-related adaptations rather than relying solely on the frequency of reported findings and is consistent with the Synthesis Without Meta-analysis (SWiM) reporting recommendations [24].

### 2.6. Synthesis of Results

A narrative synthesis approach was employed to integrate findings across studies. Results were synthesized according to major biomechanical domains, including spatiotemporal parameters, joint kinematics, joint kinetics, stiffness and spring-mass behaviour, impact loading, coordination and variability, inter-limb asymmetry, and balance-related measures. This domain-based synthesis enabled the identification of common fatigue-related biomechanical patterns while accounting for methodological and outcome heterogeneity across studies. The synthesis process involved grouping studies according to fatigue modality, running environment, and biomechanical outcome domain, followed by structured comparison of directional trends and consistency of fatigue-related changes across studies. Patterns were identified within and across biomechanical domains (e.g., spatiotemporal parameters, joint kinetics, stiffness, and coordination), while discrepancies were interpreted in relation to differences in fatigue intensity, participant characteristics, and measurement techniques. This approach followed established methodological guidance for narrative synthesis in systematic reviews of complex and heterogeneous interventions [24,25].

### 2.7. Data Synthesis

Data synthesis emphasized qualitative integration rather than statistical pooling. Studies were grouped based on similarities in fatigue modality, running environment, and biomechanical outcome domain. Consistent trends across studies were highlighted, while conflicting findings were interpreted in the context of differences in participant characteristics, fatigue intensity, and measurement techniques. Statistical integration was inappropriate due to substantial heterogeneity in fatigue protocols (sprint vs. endurance), running environments, participant characteristics, and biomechanical outcome definitions across studies. Key variables such as ankle stiffness, joint kinetics, and spatiotemporal parameters were quantified using non-comparable models, speeds, and fatigue thresholds. Accordingly, a narrative synthesis was adopted to preserve contextual specificity and enable mechanistic interpretation, consistent with recommendations for synthesis without meta-analysis in heterogeneous biomechanics research [24,25,26].

### 2.8. Additional Analyses and Publication Bias

Formal assessment of publication bias using funnel plots or regression-based methods was not conducted, as such approaches are not recommended when meta-analysis is not performed or when substantial methodological and clinical heterogeneity exists among included studies [18]. Given the diversity of fatigue protocols, participant populations, and biomechanical outcome measures, quantitative evaluation of small-study effects was deemed inappropriate. Instead, potential publication bias was addressed qualitatively through comprehensive searches across multiple electronic databases, screening of the reference lists of eligible articles, and consideration of grey literature sources, such as conference proceedings and academic theses, where available. This approach aligns with recommendations for narrative and qualitative evidence synthesis, which emphasize comprehensive evidence capture and contextual interpretation over statistical assessment of bias [27]. In addition, the influence of methodological quality, study design, and sample size on reported biomechanical findings was carefully considered during data interpretation to mitigate the impact of selective reporting.

## 3. Results

Across the 24 included studies, the consistency of fatigue-related adaptations varied across biomechanical domains. High consistency was observed for increased ground contact time, reduced ankle power and stiffness, elevated impact loading, and increased coordination variability. Moderate consistency was evident for changes in joint range of motion, proximal joint loading, and inter-limb asymmetry. Lower consistency was observed for cadence adjustments and ankle range of motion changes, reflecting protocol and population variability. 

This systematic literature search and study selection process identified 24 studies published between 2010 and 2025 that met the predefined inclusion criteria. At the full-text screening stage, an additional 23 studies were excluded because they examined fatigue without reporting biomechanical outcomes or assessed biomechanics without an explicit fatigue protocol, highlighting the fragmented nature of the existing literature. These studies collectively examined the biomechanical effects of exercise-induced fatigue during running across a wide range of participant populations, fatigue modalities, and experimental environments. The results are presented in a structured manner, beginning with an overview of study characteristics and methodological quality, followed by a domain-wise synthesis of fatigue-related biomechanical adaptations, including spatiotemporal parameters, joint kinematics and kinetics, spring-mass behaviour, impact loading, coordination variability, neuromuscular indicators, and inter-limb asymmetry. Findings are summarized descriptively to highlight the direction, consistency, and injury relevance of biomechanical changes observed under fatigued conditions.

Table 2 summarizes 24 experimental and field-based studies (2010–2025) examining fatigue-related biomechanical changes during running and jumping tasks across recreational, trained, and clinical runner populations. Fatigue was induced using diverse protocols, including repeated sprints, graded treadmill running, endurance races, and sport-specific tasks. Outcome domains covered spring–mass mechanics, joint kinematics and kinetics, impact loading, coordination, variability, symmetry, balance, and bone strain.

Methods included force plates, motion capture, IMUs, EMG, and musculoskeletal–finite element modelling. Despite heterogeneity, consistent patterns emerged, including increased contact time, altered or redistributed stiffness, elevated joint loading, and greater movement variability under fatigue. Several studies showed that performance outputs were preserved despite substantial internal biomechanical alterations. Overall, the findings indicate that fatigue induces multi-system biomechanical adaptations that may increase musculoskeletal injury risk.

Table 3 presents the methodological quality of included studies assessed using the RoB-2 tool. Most studies were rated as having “some concerns, mainly due to non-randomized or within-subject designs. Outcome measurement and missing data domains were generally rated as low risk. A limited number of studies demonstrated a high overall risk of bias.

Table 4 summarizes fatigue intervention characteristics across the 24 included studies, with fatigue primarily conceptualized as an acute exposure induced through laboratory protocols or occurring during real-world endurance events. Fatigue modalities ranged from short-duration maximal tasks to prolonged continuous running and competitive races. Termination criteria included volitional exhaustion, fixed task completion, or race distance, while fatigue monitoring relied on performance decline, physiological markers, perceptual scales, or wearable-derived metrics.

Although intervention designs were heterogeneous, consistent biomechanical responses emerged, including increased joint loading, altered stiffness regulation, elevated impact metrics, and greater movement variability. Several studies also demonstrated task-dependent or group-specific vulnerability, highlighting that fatigue effects are context- and population-sensitive. Collectively, the interventions produced mechanical adaptations consistent with elevated musculoskeletal loading and potential injury risk.

Table 5 synthesises common fatigue protocol characteristics and methodological patterns across the included studies. Fatigue was almost exclusively conceptualised as an acute exposure, with biomechanical assessments conducted immediately following fatigue induction, and no studies examining chronic or cumulative fatigue effects. Continuous running was the dominant fatigue modality, while fewer studies employed sprint-based, endurance race, or task-transfer designs. Most protocols were laboratory-based treadmill interventions, with a smaller number of field studies capturing fatigue during real-world races, improving ecological validity but reducing experimental control. 

Fatigue duration and intensity varied widely, ranging from brief maximal tasks to prolonged endurance exposures, and termination criteria were inconsistently defined, often relying on task completion or volitional exhaustion without physiological thresholds. Fatigue verification methods were heterogeneous, including subjective ratings, mechanical performance decrements, and wearable-derived metrics, and recovery allowances were rarely incorporated before biomechanical testing. Collectively, the substantial protocol heterogeneity limited quantitative pooling of results and supported the use of narrative synthesis.

Table 6 summarises the direction, consistency, and injury-relevant nature of biomechanical changes observed under fatigue. Across studies, fatigue was most consistently associated with increased ground contact time, reduced distal joint contribution (notably decreased ankle power and functional stiffness), and a compensatory shift toward greater knee and hip loading. Impact-related measures, including tibial acceleration and vertical loading rate, tended to increase during prolonged or high-speed conditions, indicating elevated skeletal loading. 

Fatigue also produced marked increases in coordination and motor variability, particularly in trunk–pelvis–hip coupling, suggesting reduced neuromuscular stability. Several studies reported increased inter-limb asymmetry and impaired balance or landing stability, especially following task-transfer protocols. While the magnitude of individual biomechanical responses varied across studies and populations, the overall pattern indicates a shift toward less efficient and more injury-relevant mechanical strategies under fatigue.

**Figure 2:**
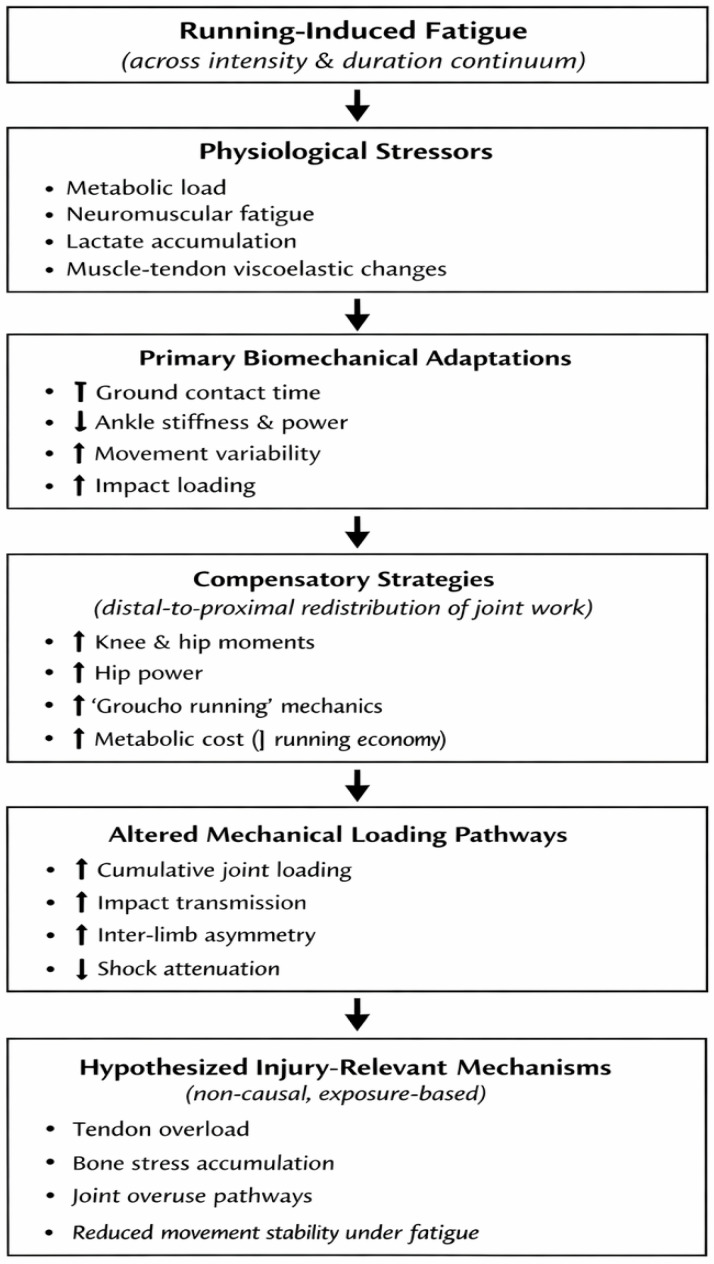
Conceptual framework linking running-induced fatigue, biomechanical adaptations, and injury-relevant mechanical pathways.

This schematic illustrates (Figure 2) how running-induced fatigue, arising across a continuum of intensity and duration, initiates physiological stressors that drive progressive biomechanical adaptations. These adaptations include alterations in spatiotemporal parameters, joint mechanics, stiffness, impact loading, and coordination. Compensatory strategies, particularly distal-to-proximal redistribution of joint work, may help preserve running performance but increase metabolic cost and modify mechanical loading patterns. The framework highlights hypothesized injury-relevant mechanical pathways rather than direct injury causation, as most included studies did not prospectively assess injury outcomes.

## 4. Discussion

### 4.1. Overview of Main Findings

The present systematic review synthesized evidence from 24 original studies published between 2010 and 2025 to examine the effects of fatigue on lower-limb biomechanics during running. Collectively, the findings demonstrate that exercise-induced fatigue consistently alters spatiotemporal parameters, joint kinematics and kinetics, spring-mass behaviour, impact loading, coordination variability, and inter-limb symmetry. Although methodological heterogeneity precluded quantitative pooling, strong qualitative convergence was evident across multiple biomechanical domains, suggesting that fatigue induces systematic and potentially injury-relevant mechanical adaptations during running (see Table 2, Table 4 and Table 6). Rather than reiterating individual findings already summarized in the results and tables, the following discussion focuses on the underlying mechanisms and functional implications of fatigue-induced biomechanical adaptations. Specifically, emphasis is placed on why these adaptations emerge under fatigue and how they influence running performance, mechanical efficiency, and potential injury-relevant loading pathways.

### 4.2. Effects of Fatigue on Spatiotemporal Parameters

One of the most consistent fatigue-related adaptations across the included literature involved spatiotemporal parameters, particularly changes in stance-phase mechanics (Table 6). The observed increase in ground contact time under fatigue likely reflects reduced neuromuscular efficiency and slower force application, indicating a diminished capacity to rapidly generate and absorb forces during stance [7,8,38]. This interpretation is supported by force–time impairments reported in countermovement jump and running fatigue protocols, which demonstrate fatigue-related reductions in rate of force development and impulse generation [5]. While subtle adjustments in cadence and stride length may serve as compensatory strategies to preserve running speed, prolonged contact times increase cumulative musculoskeletal loading per stride and modify impact attenuation characteristics. Consequently, sustained or repeated exposure to these altered loading patterns has been associated with theoretical mechanisms underlying overuse-related musculoskeletal injury development [6,40,41,42].

### 4.3. Joint Kinematic Adaptations Under Fatigue

Joint kinematic adaptations to fatigue were consistently observed at the ankle, knee, and hip (Table 6). Increased knee flexion during stance was one of the most frequently reported changes, particularly in novice and recreational runners [10,15,28]. While greater knee flexion may enhance shock absorption under fatigued conditions, it simultaneously increases patellofemoral joint stress, potentially contributing to knee-related overuse injuries. In parallel, several studies identified increased ankle dorsiflexion and altered ankle range of motion following fatigue [14,17]. These changes suggest reduced elastic recoil capacity of the ankle–Achilles complex, which may shift mechanical demands proximally to the knee and hip joints. This proximal redistribution of load is further supported by evidence of increased hip flexion and hip joint work under fatigued conditions [10,36].

### 4.4. Joint Kinetic Alterations and Proximal Load Redistribution

Fatigue-induced changes in joint kinetics further reinforce a proximal shift in mechanical demand during running. Runner expertise is an important factor when interpreting these adaptations. Novice and recreational runners typically exhibit larger kinematic deviations, increased movement variability, and greater stiffness loss under fatigue. In contrast, trained and elite runners often maintain more stable mechanics and distal joint contribution, reflecting superior neuromuscular coordination and fatigue resistance. Accordingly, findings should be interpreted with caution when performance levels are blended.

Reductions in ankle joint power and stiffness were consistently reported across laboratory- and field-based studies [8,9,11] (See Table 2 and Table 6). Given the ankle’s critical role in elastic energy storage during running, fatigue-related impairment at this joint promotes a distal-to-proximal redistribution of mechanical work. This redistribution is characterized by reduced ankle contribution and compensatory increases in knee joint moments and hip power output [10,31,43,44]. While this strategy may help preserve running speed under fatigued conditions, it increases reliance on active muscular work at proximal joints and reduces elastic energy return. This compensation pattern resembles a “groucho running” strategy and is therefore likely accompanied by an increased metabolic cost of transport and reduced running economy.

When such compensatory loading is sustained or repeatedly exposed under fatigue, it may contribute to injury-relevant mechanical loading pathways at the knee, hip, and lumbar regions. These pathways should be interpreted as theoretical rather than direct injury causation [43,45].

Fatigue-related biomechanical adaptations should not be interpreted as abrupt transitions between non-fatigued and fatigued states. Instead, available evidence indicates that mechanical adjustments emerge progressively over time. Early fatigue is often characterized by subtle kinematic changes, such as increased joint range of motion and prolonged ground contact time, reflecting initial neuromuscular compensation. As fatigue accumulates, reductions in ankle joint power and stiffness become more pronounced, compromising elastic energy storage and push-off capacity. In later stages, runners increasingly adopt proximal compensatory strategies, marked by elevated knee and hip joint moments and power output. This phased adaptation highlights fatigue as a dynamic process in which biomechanical strategies evolve to maintain task performance despite declining neuromuscular capacity. Sex, training status, and age were inconsistently reported across studies, limiting subgroup interpretation; most samples were male or mixed-sex, with few sex-specific analyses and minimal representation of youth or older runners. Available evidence indicates that novice runners exhibit greater kinematic deviations and stiffness loss under fatigue than trained or elite runners, highlighting the need for future stratified investigations.

### 4.5. Spring-Mass Behaviour and Mechanical Efficiency

Spring-mass behaviour emerged as a central theme across the included literature. Multiple studies demonstrated reductions in vertical and leg stiffness following fatigue (Table 2 and Table 6), irrespective of running experience or fatigue modality [7,9,11,12]. Reduced stiffness compromises the efficiency of elastic energy storage and return, which may partly explain the observed increases in ground contact time and joint range of motion. From an injury-mechanics perspective, diminished stiffness reduces the system’s ability to attenuate impact forces, thereby increasing the transmission of loads to passive structures such as bone and cartilage. This interpretation is consistent with findings from ultra-endurance and speed-perturbation studies reporting increased tibial acceleration and strain under fatigued conditions [13,35]. Importantly, reductions in global leg and vertical stiffness under fatigue should not be interpreted solely as centrally mediated or coordinative strategies. Acute fatigue also induces tissue-level alterations in the viscoelastic properties of the muscle tendon unit, including changes in tendon compliance, muscle stiffness, and damping characteristics. Exhaustive or prolonged running has been shown to produce heterogeneous reductions in tendon stiffness and alterations in muscle force–length behavior, which directly impair elastic energy storage and return. These acute viscoelastic changes provide a mechanistic explanation for the observed increases in ground contact time, joint excursion, and impact transmission under fatigued conditions. Accordingly, fatigue-related reductions in spring–mass stiffness likely reflect the combined influence of neuromuscular control strategies and transient alterations in muscle tendon mechanical properties.

### 4.6. Impact Loading and Injury-Relevant Mechanical Indicators

Impact loading variables provide further insight into fatigue-related injury mechanisms (Table 6). Several studies reported increased vertical loading rates and tibial acceleration following fatigue [13,14,38,46]. Elevated loading rates are widely regarded as biomechanical indicators that may contribute to stress-related injury mechanisms, particularly when combined with prolonged exposure and insufficient recovery; however, most studies included in this review did not prospectively assess injury incidence. Importantly, these impact-related changes were observed not only in laboratory treadmill studies but also under real-world conditions, such as ultramarathon and half-marathon running [13,38]. This convergence across experimental and field environments strengthens the ecological validity of fatigue-induced biomechanical adaptations. Recent systematic evidence highlights the complexity of linking biomechanical deviations to injury outcomes. No single lower-limb biomechanical variable has been identified as an independent causal factor for groin pain [46], supporting the present findings that fatigue induces distributed, multi-joint mechanical adaptations rather than isolated injury-specific patterns [47].

### 4.7. Mechanical Correlates Versus Direct Injury Outcomes

It is important to distinguish between biomechanical variables that are mechanically associated with injury risk and direct clinical injury outcomes. The majority of studies included in this review quantified mechanical indicators such as loading rates, joint moments, stiffness, and movement variability, but did not prospectively track injury incidence. Accordingly, these variables should be interpreted as biomechanical correlates or theoretical pathways that may contribute to injury development rather than causal predictors of injury. While established injury biomechanics literature supports the relevance of these mechanical markers, definitive injury risk inference requires longitudinal designs integrating fatigue exposure, biomechanical monitoring, and injury surveillance. This distinction is essential to avoid over-interpretation of fatigue-related biomechanical adaptations as direct evidence of injury causation.

### 4.8. Coordination Variability and Motor Control Under Fatigue

Coordination variability and motor control adaptations represent another important dimension of fatigue-related biomechanical change (Table 2 and Table 6). Increased coordination variability at the trunk–pelvis–hip complex was consistently reported following fatigue, particularly at higher running speeds [16]. Similarly, fatigue-induced alterations in the structure of motor variability have been demonstrated in novice runners [15], indicating reduced movement stability [15]. While some degree of variability is essential for adaptive movement, excessive or poorly structured variability under fatigue may reflect compromised neuromuscular control and reduced capacity to respond to perturbations, thereby increasing non-contact injury risk during dynamic running conditions.

### 4.9. Inter-Limb Asymmetry and Bilateral Load Distribution

Inter-limb asymmetry emerged as a notable fatigue-related adaptation across several studies (Table 6). Increased asymmetry in joint moments following prolonged running-induced fatigue has been reported, suggesting uneven load distribution between limbs [17]. Such asymmetry may arise from unilateral strength deficits, neuromuscular fatigue, or compensatory strategies and has been implicated in the development of unilateral overuse injuries. These findings underscore the importance of assessing bilateral mechanics when evaluating fatigue effects, particularly in runners with pre-existing asymmetries or injury history. Injury-prediction evidence further supports the injury relevance of fatigue-related biomechanical asymmetries. From an injury epidemiology perspective, fatigue-induced inter-limb asymmetry represents a meaningful mechanical exposure that alters tissue loading patterns and interacts with individual susceptibility, contributing to injury risk when asymmetrical loading is repeated over time [48]. Although derived from a jumping-dominant task, these findings closely align with fatigue-induced increases in asymmetry, coordination variability, and proximal loading reported in running, reinforcing their injury relevance across high-impact activities. Interpretation of fatigue-related inter-limb asymmetry should extend beyond unilateral strength deficits to include the physiological dynamics of high-intensity exercise. During repeated maximal efforts, elevated blood lactate has been shown to modulate muscle activation patterns and intermuscular coordination, which can alter asymmetry expression under fatigue. Recent evidence from elite sprinters demonstrates that progressive lactate accumulation during repeated sprinting is associated with systematic changes in lower-limb muscle activity and, in some cases, reduced inter-limb asymmetry, indicating a fatigue-induced reorganization of neuromuscular control rather than purely mechanical imbalance [49].

### 4.10. Neuromuscular Performance Degradation

Neuromuscular indicators derived from force–time analysis further support the presence of fatigue-induced mechanical degradation (Table 2 and Table 6). Wu et al. (2019) [5], along with Gathercole et al. (2015) and McMahon et al. (2018), demonstrated that countermovement jump force time signatures can distinguish between neuromuscular and metabolic fatigue, with fatigue states characterized by reductions in impulse and rate of force development [5,45,50]. These impairments likely contribute to the observed reductions in lower-limb stiffness and joint power during running, as diminished force-generating capacity compromises effective energy transfer across the muscle tendon units. Importantly, such neuromuscular deficits may persist beyond the immediate fatigue task, resulting in altered movement strategies and impaired load attenuation during subsequent training or competition sessions, thereby increasing susceptibility to overuse and non-contact injuries [50,51,52].

### 4.11. Transfer Effects to Functional Tasks

Task-transfer studies provided additional insight into how running-induced fatigue influences biomechanical performance in related tasks (Table 6). Several investigations reported impaired landing stability and balance performance following fatigue, as evidenced by altered joint loading during tuck-jump assessments and Y-Balance tests [34,37,53]. These findings indicate that fatigue effects extend beyond steady-state running to dynamic tasks requiring rapid force production and postural control, which is particularly relevant for sports involving combined running, jumping, and cutting actions.

### 4.12. Methodological Heterogeneity and Research Gaps

Despite the overall consistency of directional trends, considerable heterogeneity was observed across studies in fatigue protocols, participant characteristics, and measurement techniques. Fatigue duration ranged from short, task-induced protocols to extreme ultra-endurance events, and verification methods varied from subjective measures such as RPE to objective performance or force-based criteria. This variability limits direct comparison across studies and highlights the need for standardized fatigue definitions and verification procedures in future biomechanical research. Nonetheless, the convergence of findings across diverse methodologies suggests that the fundamental biomechanical consequences of fatigue are robust.

### 4.13. Practical Implications

From a practical perspective, the findings of this review have important implications for training, injury prevention, and rehabilitation. Coaches and practitioners should recognize that fatigue alters not only performance outcomes but also underlying mechanical loading patterns. Training interventions emphasizing fatigue-resistant strategies such as improving ankle stiffness, proximal joint control, and neuromuscular coordination may help mitigate adverse biomechanical adaptations. Additionally, wearable sensor technologies demonstrated in several included studies [38,39] offer promising tools for real-time fatigue monitoring and individualized load management. From a practical perspective, the findings of this review support the use of targeted monitoring and training strategies to manage fatigue-related biomechanical alterations. For example, wearable inertial measurement units (IMUs) or instrumented treadmills can be used to monitor changes in ground contact time, stride characteristics, and impact loading as indicators of emerging fatigue during training or competition. Reductions in ankle stiffness or push-off power may signal the need for load modification, recovery emphasis, or technique-focused interventions. Strength and conditioning programs may prioritize ankle–foot complex stiffness, calf muscle endurance, and elastic energy utilization to delay distal fatigue and reduce compensatory proximal loading. Additionally, coaches may employ neuromuscular fatigue monitoring tools such as countermovement jump force–time analysis or asymmetry metrics to inform individualized training adjustments and recovery strategies, particularly during high-intensity or prolonged running blocks. For example, during a rehabilitation or gait retraining session using virtual reality (VR), a therapist could integrate wearable inertial sensors placed on the shank or foot to continuously monitor ground contact time, step symmetry, and impact loading in real time. If the system detects progressive increases in contact time asymmetry or excessive impact peaks as fatigue develops, the VR environment can provide immediate visual or auditory feedback prompting gait correction, pacing adjustment, or rest intervals. Such sensor-guided feedback allows clinicians to individualize load progression, minimize compensatory movement patterns, and enhance neuromuscular control during immersive training or rehabilitation sessions.

### 4.14. Limitations and Future Directions

Several limitations of the current evidence base warrant consideration. Most included studies employed small sample sizes and laboratory-based designs, which may limit generalizability. Moreover, few studies directly linked biomechanical changes to injury outcomes, necessitating cautious interpretation of injury relevance. Nevertheless, the mechanical indicators identified in this review are well supported by established injury biomechanics literature [6] and provide a strong foundation for hypothesis generation in future longitudinal investigations.

An important limitation of the present review is the aggregation of biomechanical outcomes across fatigue protocols that arise from fundamentally different physiological mechanisms. Short-duration, high-intensity running (e.g., sprint or middle-distance efforts) is predominantly characterized by metabolic acidosis, inorganic phosphate accumulation, and rapid neuromuscular fatigue, whereas prolonged endurance running involves distinct processes such as glycogen depletion, muscle damage, and central fatigue. Although similar biomechanical adaptations, such as reduced ankle stiffness or increased joint flexion, were observed across fatigue modalities, these adaptations may emerge from different underlying physiological states. Accordingly, the term ‘fatigued state’ in this review should be interpreted as a functional biomechanical condition rather than a uniform physiological entity, and caution is warranted when extrapolating findings across sprint- and endurance-based fatigue contexts. Another important limitation is that the injury relevance of fatigue-induced biomechanical adaptations was interpreted indirectly. The majority of included studies quantified biomechanical variables without prospectively tracking injury outcomes. Consequently, references to injury risk throughout this review should be interpreted as hypothesized or theoretical mechanical pathways informed by established injury biomechanics literature, rather than as direct evidence of injury causation. Future longitudinal studies integrating fatigue exposure, biomechanical assessment, and injury surveillance are required to confirm these relationships. Furthermore, substantial heterogeneity in fatigue protocols, including differences in fatigue modality, duration, intensity, termination criteria, and verification methods, limits the direct comparability of biomechanical outcomes across studies. Variations in experimental design may influence both the magnitude and temporal expression of fatigue-related adaptations, contributing to between-study variability. This heterogeneity constrained the ability to quantitatively synthesize findings and necessitated a narrative, domain-based approach. Consequently, observed consistencies should be interpreted as indicative of general biomechanical trends rather than precise estimates of fatigue effects across all running contexts.

## 5. Conclusions

This systematic review demonstrates that exercise-induced fatigue consistently alters lower-limb biomechanics during running, affecting spatiotemporal characteristics, joint kinematics and kinetics, spring-mass behavior, impact loading, neuromuscular output, coordination variability, and inter-limb symmetry. Across studies published between 2010 and 2025, fatigue-related biomechanical adaptations followed clear directional trends despite heterogeneity in fatigue protocols, participant populations, and measurement techniques, indicating robust and systematic mechanical responses to fatigue. Fatigue was commonly associated with prolonged ground contact time, reduced ankle joint power and stiffness, increased joint range of motion, elevated impact loading, and greater movement variability. These changes reflect a decline in mechanical efficiency and a redistribution of load from distal to proximal joints, particularly toward the knee and hip. Such adaptations may help maintain running performance in the short term but simultaneously create mechanical conditions associated with increased susceptibility to overuse and non-contact injuries, especially during prolonged or repeated exposure to fatigue. Importantly, similar biomechanical patterns were observed across controlled laboratory studies and real-world endurance running conditions, supporting the ecological validity and practical relevance of the findings. Transfer effects to functional tasks such as jumping and balance assessments further indicate that fatigue-related biomechanical deficits extend beyond steady-state running and may influence movement stability in sport-specific contexts. Collectively, these findings highlight fatigue as a critical determinant of running biomechanics and injury-relevant mechanical loading. Integrating fatigue-aware biomechanical assessment, neuromuscular conditioning, and individualized load management into training and rehabilitation programs may help mitigate adverse fatigue-related adaptations. Future research should prioritize standardized fatigue protocols, longitudinal designs linking biomechanics to injury outcomes, and wider application of wearable technologies to advance understanding of fatigue–biomechanics–injury relationships. From an applied perspective, the most actionable fatigue-related biomechanical adaptations include prolonged ground contact time, reductions in ankle stiffness and push-off power, elevated impact loading, and increased coordination variability. These variables are consistently observable across protocols and can be monitored using wearable sensors, force platforms, or field-based performance testing. Targeting these adaptations through load management, ankle–foot complex conditioning, neuromuscular control training, and recovery optimization may offer practical benefits for injury risk mitigation and performance sustainability.

## Figures and Tables

**Table 1:** Inclusion and Exclusion Criteria for Study Selection.

Domain	Inclusion Criteria	Exclusion Criteria
Study design	Original empirical research, including experimental, quasi-experimental, observational, and field-based biomechanical studies	Systematic reviews, meta-analyses, scoping reviews, bibliometric analyses, narrative reviews, editorials, commentaries
Publication period	Studies published between January 2010 and December 2025	Studies published before 2010
Population	Human participants engaged in running or running-related tasks (recreational, trained, elite, youth, or clinical subgroups)	Animal studies; non-running populations (e.g., cycling-only, walking-only, resistance-training-only studies)
Age group	Youth, adolescent, and adult participants	Studies exclusively involving children with pathological gait unrelated to fatigue
Fatigue exposure	Studies that explicitly induced or quantified fatigue, including running-induced fatigue, sprint-induced fatigue, prolonged running, or task-induced fatigue with relevance to running biomechanics	Studies without a defined fatigue protocol or without pre- vs. post-fatigue biomechanical comparison
Primary outcome focus	Lower-limb biomechanics, including kinematics, kinetics, stiffness, impact loading, coordination, variability, asymmetry, or neuromuscular mechanical outcomes	Studies reporting only physiological (e.g., VO_2_max), metabolic, perceptual, or psychological outcomes without biomechanical measures
Biomechanical measures	Quantitative biomechanical data derived from motion capture, force plates, instrumented treadmills, IMUs, accelerometers, pressure sensors, or validated musculoskeletal models	Qualitative assessments, self-report measures, or clinical scores without biomechanical quantification
Movement context	Running performed on treadmill, overground, track, field, or simulated competition settings	Non-running movement contexts (e.g., cycling, swimming, resistance exercise) without a running component
Transfer tasks	Studies assessing transfer effects of running-induced fatigue on related biomechanical tasks (e.g., countermovement jump, landing, balance tests)	Task-based fatigue studies unrelated to running (e.g., upper-limb fatigue only)
Outcome relevance	Outcomes relevant to functional morphology, movement mechanics, performance adaptation, or injury-related mechanical loading	Studies focusing solely on performance time or success without biomechanical explanation
Instrumentation quality	Use of validated biomechanical instrumentation with clearly described measurement protocols	Use of non-validated devices or insufficient description of biomechanical methods
Language	Articles published in English	Non-English publications
Accessibility	Full-text articles available	Abstract-only publications with insufficient methodological detail

**Table 2:** Characteristics of Included Studies (2010–2025).

Author & Year	Study Design	Participants	Sample (Sex; n)	Fatigue Protocol/Task	Primary Outcome Domain	Biomechanical Measures	Instruments	Main Findings (Fatigue Effect)
Girard et al. (2011) [9]	Experimental (repeated-sprint)	Physically active recreational athletes	Male; n = 16	12 × 40 m maximal running sprints with 30 s passive recovery	Spring–mass mechanics and stride characteristics	Vertical stiffness (Kvert), leg stiffness (Kleg), contact time, flight time, stride frequency, stride length, braking and push-off forces	5-m force-plate system (GRF) + radar speed system	Vertical stiffness significantly decreased across sprints; leg stiffness showed no significant change; contact, flight, and swing times increased; stride frequency and push-off force decreased; center of mass vertical displacement increased
Girard et al. (2013) [7]	Experimental (time-trial, repeated measures)	Competitive triathletes	Male; n = 12	5-km self-paced running time trial on indoor track	Running mechanics and spring–mass behavior	Kvert, Kleg, contact time, stride length, stride frequency, peak vertical force, braking and push-off forces, CM displacement	5-m force-plate system (GRF) + radar speed system	Vertical stiffness decreased (~6%); leg stiffness remained unchanged; contact time and total stride duration increased; stride length and frequency decreased; peak vertical and braking forces decreased; CM vertical displacement showed no significant change
Koblbauer et al. (2014) [28]	Repeated-measures	Novice runners	Mixed; n = 17	Borg-controlled run to fatigue	Joint kinematics	Trunk flexion, ankle eversion	Motion capture	Increased trunk flexion and ankle eversion after fatigue
Fischer et al. (2015) [11]	Experimental (repeated measures)	Recreational runners	Mixed; n = 11	60-s maximal counter-movement jump fatigue followed by overground running at different speeds	Spring–mass mechanics and spatiotemporal parameters	Kvert, COM displacement (ΔZ), peak vertical force, step frequency, step length, aerial time	Force plate integrated in track + 2D video	No significant change in vertical stiffness; COM vertical displacement decreased; step frequency increased and step length decreased; peak vertical force decreased under fatigue
Morin et al. (2015) [29]	Experimental (laboratory)	Physically active males including sprint- and team-sport athletes	Male; n = 14	Single 6-s maximal sprint acceleration on instrumented treadmill	Sprint acceleration kinetics and neuromuscular correlates	Horizontal (FH), vertical (FV), resultant GRF, sprint velocity, EMG of BF, RF, VL, Glut	Instrumented treadmill (force transducers), surface EMG, 2D motion analysis	Greater horizontal GRF was significantly associated with higher biceps femoris EMG during end-of-swing and greater eccentric hamstring torque; vertical GRF was not related to sprint performance; study did not involve fatigue comparison
Giandolini et al. (2016) [13]	Field experimental (pre–post race)	Experienced ultramarathon runners	Mixed; n = 23	110-km mountain ultramarathon (UTMB) with pre- and post-race treadmill testing	Impact biomechanics and lower-limb kinematics	Peak tibial acceleration, impact frequency content, step frequency, ankle ROM, foot, ankle and tibial angles at contact	Tibial accelerometers, 2D video analysis, treadmill	Step frequency increased (~+2.7%) and ankle ROM decreased (~−4.1%) after the race; impact acceleration magnitude did not change significantly; runners showed a tendency toward flatter foot strike patterns, particularly in non-rearfoot strikers, consistent with protective fatigue-related adaptations
Basile et al. (2017) [30]	Experimental (pre–, post–, pilot)	Youth distance runners (12–14 yrs)	Mixed; n = 4	Prolonged treadmill running at ~70% VO_2_max with data at start, mid, and end	Impact kinetics and limb acceleration	Peak vertical GRF, heel acceleration, cadence, step length, strike pattern	Instrumented treadmill (dual force plates), 3D motion capture (Vicon)	Fatigue-related changes were highly individual; some runners showed increased peak vertical GRF and development of heel-strike transient, while cadence and step length showed no consistent group-level change.
Wu et al. (2019) [5]	Randomized crossover, repeated measures	Recreational athletes	Mixed; n = 10	Repeated sprint training sessions (low, moderate, high workload) with CMJ testing pre, post, and up to 48 h	Neuromuscular and metabolic fatigue signatures	CMJ concentric force-time variables (relPeakF, relPeakP, concentric time, time to peak force)	Portable force plate (600 Hz)	PCA and fPCA identified distinct fatigue signatures, with metabolic fatigue dominating ≤1 h post-exercise and neuromuscular fatigue evident from 3–48 h; CMJ force-time profiles successfully predicted fatigue state
Riazati et al. (2020) [31]	Crossover experimental	Master class runners	Mixed; n = 20	Energy-expenditure matched HIIT (6 × 800 m) vs. medium-intensity continuous run (MICR)	Muscle strength, gait kinematics, and running variability	Hip and knee isometric strength, sagittal and frontal plane joint angles, coordination variability (CRP, CAV), spatiotemporal parameters	Hand-held dynamometry; 3D motion capture (Vicon)	Significant reductions in hip and knee strength occurred after both run types; hip frontal and sagittal ROM increased, while knee kinematics showed no significant group-level changes; running coordination variability increased, with individual-level analysis indicating greater fatigue effects following HIIT
Yu et al. (2020) [32]	Experimental (pre–post)	Physically active novice runners	Male; n = 15	Progressive treadmill running to volitional exhaustion followed immediately by CMJ testing	Jump biomechanics (kinematics and kinetics)	Joint angles and ROM (ankle, knee, hip), joint moments, peak vertical GRF, jump height	3D motion capture (Vicon), force platform (Kistler)	Lower-limb joint kinematics and moments changed significantly after fatigue, while jump height and peak vertical GRF remained unchanged.
Möhler et al. (2021) [8]	Experimental (repeated measures, treadmill)	Expert middle-distance runners	Male; n = 13	Constant-speed middle-distance treadmill run at individual fatigue speed until exhaustion (~4 min)	Running mechanics, joint kinematics, and stiffness	Spatiotemporal parameters, leg and vertical stiffness, 3D joint kinematics, COM displacement, ROM	3D motion capture (Vicon); stiffness estimated from kinematics (no force plates)	Stance time increased and both leg and vertical stiffness decreased, with greater joint ROM and reduced vertical COM displacement under fatigue.
Yu et al. (2021) [10]	Experimental (pre post)	Novice runners	Male; n = 15	Progressive treadmill running to volitional exhaustion	Joint kinetics (3 planes)	Hip, knee, ankle joint moments and powers	3D motion capture (Vicon), force platform	Joint moments and powers increased at the ankle, knee, and hip, especially in the frontal and transverse planes, indicating higher joint loading after fatigue.
Chalitsios et al. (2022) [33]	Experimental (cross-sectional with ML classification)	Recreational runners	Mixed; n = 13	Exhaustive incremental treadmill run to ventilatory threshold and exhaustion	Movement variability & fatigue sensitivity	Trunk angular range (AP & frontal), GRF loading rate, coordination features	Instrumented treadmill, motion capture	Trunk frontal and AP angular ranges and GRF loading rate were the most sensitive indicators of fatigue; kinetic variability increased non-linearly under fatigue.
Encarnación-Martínez et al. (2022) [14]	Experimental (crossover)	Recreational runners	Male; n = 18	Central fatigue (30-min run at 85% MAS) vs. peripheral fatigue (isokinetic quadriceps–hamstrings) followed by treadmill running	Impact transmission	Tibial and head acceleration, shock attenuation (time & frequency domain)	IMUs (tibia, head), treadmill	Central fatigue increased high-frequency tibial impact power and shock attenuation, whereas peripheral fatigue produced no significant impact changes.
Gao et al. (2022) [17]	Experimental (pre post)	Amateur runners	Male; n = 18	Running-induced fatigue protocol followed by overground running	Gait symmetry	Symmetry angles of hip, knee, and ankle joint angles, moments, and stiffness (3 planes)	3D motion capture (Vicon), force plates	Fatigue increased asymmetry in knee and hip joint moments and angles, particularly in coronal and transverse planes.
Möhler et al. (2022) [15]	Experimental (pre–post, treadmill)	Novice runners	Male; n = 14	Prolonged treadmill running to exhaustion	Motor variability and CoM control	UCM-based variability of joint configurations stabilising CoM trajectory	3D motion capture	Step-to-step motor variability increased, and control of the centre of mass decreased, while overall CoM stability was preserved.
Munoz (2022) [12]	Experimental (pre -post)	ROTC cadets	Mixed; n = 16	Graded exercise test followed by exhaustive treadmill run; overground running analysis	Stiffness modulation	Vertical (Kvert), leg (Kleg), and joint stiffness (ankle, knee, hip)	3D motion capture, force plates	Group means of Kvert and Kleg did not change; runners redistributed stiffness across joints, with increased knee moments and hip excursion under fatigue.
Khaleghi Tazji et al. (2023) [16]	Quasi-experimental (pre–post, repeated measures)	Recreational runners	Mixed; n = 24	Incremental treadmill fatigue (Borg-based) with running at preferred, 80%, and 120% speed	Motor coordination	Continuous relative phase (CRP) and coordination variability (VCRP) of trunk–pelvis–hip couplings	Inertial motion capture (myoMOTION IMUs)	Fatigue reduced inter-segmental coordination and increased coordination variability, with larger effects at higher running speeds.
Huang et al. (2023) [34]	Experimental (pre–post)	Amateur athletes	Male; n = 16	Squat-based fatigue protocol followed by Y-Balance Test	Balance biomechanics	Hip, knee, ankle ROM; joint torques; joint work; COP displacement	3D motion capture (Vicon) + force plate (Kistler)	Y-Balance scores and hip/knee ROM decreased; hip torque increased (A, PL) and decreased (PM); COP displacement increased after fatigue.
Baggaley et al. (2024) [35]	Experimental modelling (repeated measures)	Recreationally active runners	Mixed; n = 17	Treadmill running at multiple grades (±10°, ±5°, 0°) and speeds (2.50–4.17 m·s^−1^)	Bone loading (fatigue–failure risk)	50th & 95th percentile tibial strain, strained volume ≥4000 µɛ	Motion capture, instrumented treadmill, musculoskeletal + finite-element models	Tibial strain and strained volume increased significantly with running speed, but not with grade, indicating higher fatigue–failure risk at faster speeds.
Jian et al. (2025) [36]	Comparative experimental (pre–post fatigue)	Female runners with and without genu valgum	Female; n = 16 (8 GV, 8 control)	Running-induced fatigue protocol on treadmill	Clinical biomechanics (ACL loading)	A-ACL and P-ACL stress and strain, knee joint stiffness, hip/knee angles	3D motion capture, force plates, OpenSim modeling, EMG validation	Fatigue significantly increased anteromedial ACL stress and reduced knee stiffness in the genu valgum group, while controls showed no significant ACL stress changes.
Kember et al. (2024) [37]	Cross-sectional experimental (pre–post fatigue)	Female netball athletes	Female; n = 12	Sport-specific fatigue protocol followed by repeated tuck-jump assessment	Jump kinetics & stabilization	Vertical GRF, COM displacement, leg stiffness, contact and flight time	Force plate, 2D video	Post-fatigue jumping became stiffer (leg stiffness ↑) with reduced jump height, COM displacement, contact and flight time, despite unchanged peak GRF.
Mitschke et al. (2025) [38]	Field experimental (repeated measures)	Endurance runners	Mixed; n = 20	Self-paced half-marathon on flat outdoor course	Field biomechanics & impact loading	Peak tibial acceleration, peak rearfoot eversion velocity, peak sagittal foot angular velocity, stride time, contact time, flight time, duty factor	IMUs (tibia, heel), HR monitor	Peak tibial acceleration, foot angular velocities, and contact time increased, while flight time decreased over the race, indicating fatigue-related impact and spatiotemporal changes.
Einicke et al. (2018) [39]	Experimental (field + ML analysis)	Adult recreational runners	Mixed; n = 22	5-km overground run to induce fatigue	Wearable biomechanics & fatigue detection	Knee and ankle kinematics, variability features, fatigue classification accuracy	IMUs, machine-learning classifiers	Fatigue-related changes in lower-limb kinematics were detectable using wearable sensors, enabling accurate fatigue state classification.

Note: Abbreviations: ACL = anterior cruciate ligament; AP = anteroposterior; BF = biceps femoris; CM = centre of mass; CMJ = countermovement jump; COP = centre of pressure; CRP = continuous relative phase; CAV = coupling angle variability; EMG = electromyography; FH = horizontal ground reaction force; fPCA = functional principal component analysis; GRF = ground reaction force; GV = genu valgum; HIIT = high-intensity interval training; IMU = inertial measurement unit; Kleg = leg stiffness; Kvert = vertical stiffness; MAS = maximal aerobic speed; MICR = medium-intensity continuous run; ML = machine learning; OpenSim = open-source musculoskeletal modelling software; PCA = principal component analysis; PL = posterolateral; PM = posteromedial; ROM = range of motion; UCM = uncontrolled manifold; UTMB = Ultra-Trail du Mont-Blanc; VCRP = variability of continuous relative phase; ΔZ = vertical centre-of-mass displacement.

**Table 3:** Risk of Bias Assessment (RoB-2) for Included Studies.

Study (Author, Year)	D1: Randomization	D2: Deviations from Intervention	D3: Missing Data	D4: Outcome Measurement	D5: Selective Reporting	Overall Risk of Bias
Girard et al. (2011) [9]	High risk	Low	Low	Low	Low	High risk
Girard et al. (2013) [7]	High risk	Low	Low	Low	Low	High risk
Koblbauer et al. (2014) [28]	High risk	Low	Low	Low	Low	High risk
Fischer et al. (2015) [11]	High risk	Low	Low	Low	Low	High risk
Morin et al. (2015) [29]	High risk	Low	Low	Low	Low	High risk
Giandolini et al. (2016) [13]	High risk	Low	Low	Low	Low	High risk
Basile et al. (2017) [30]	High risk	Some concerns	High risk (very small n)	Low	Some concerns	High risk
Wu et al. (2019) [5]	Some concerns (randomized crossover)	Low	Low	Low	Low	Some concerns
Riazati et al. (2020) [31]	Some concerns (randomized crossover)	Low	Low	Low	Low	Some concerns
Yu et al. (2020) [32]	High risk	Low	Low	Low	Low	High risk
Möhler et al. (2021) [8]	High risk	Low	Low	Low	Low	High risk
Yu et al. (2021) [10]	High risk	Low	Low	Low	Low	High risk
Chalitsios et al. (2022) [33]	High risk	Low	Low	Low	Low	High risk
Encarnación-Martínez et al. (2022) [14]	Some concerns (crossover)	Low	Low	Low	Low	Some concerns
Gao et al. (2022) [17]	High risk	Low	Low	Low	Low	High risk
Möhler et al. (2022) [15]	High risk	Low	Low	Low	Low	High risk
Munoz (2022) [12]	High risk	Some concerns	Some concerns	Low	Some concerns	High risk
Khaleghi Tazji et al. (2023) [16]	High risk	Low	Low	Low	Low	High risk
Huang et al. (2023) [34]	High risk	Low	Low	Low	Low	High risk
Baggaley et al. (2024) [35]	High risk	Low	Low	Low	Low	High risk
Jian et al. (2025) [36]	High risk	Low	Low	Low	Low	High risk
Kember et al. (2024) [37]	High risk	Low	Low	Low	Low	High risk
Mitschke et al. (2025) [38]	Some concerns (group comparison)	Low	Low	Low	Low	Some concerns
Einicke et al. (2018) [39]	High risk	Low	Low	Low	Some concerns	High risk

Note: Risk of bias was assessed using the Cochrane Risk of Bias 2 (RoB-2) tool, adapted for experimental biomechanics and kinesiology research. The following domains were evaluated: D1, bias arising from the randomization process; D2, bias due to deviations from intended interventions (fatigue protocol adherence); D3, bias due to missing outcome data; D4, bias in measurement of the outcome; and D5, bias in selection of the reported result. As most included studies employed non-randomized or within-subject experimental designs, ratings of “some concerns” for the randomization domain were common. Overall risk of bias judgments was made in accordance with Cochrane guidance. The RoB-2 tool was applied with contextual adaptation for within-subject and non-randomized experimental designs, with particular emphasis on outcome measurement validity and protocol standardization.

**Table 4:** Summary of Intervention Characteristics Across Included Studies.

Study (Author, Year)	Intervention Type (Fatigue Exposure)	Fatigue Modality	Running Environment	Fatigue Duration / Intensity	Fatigue Termination Criteria	Monitoring of Fatigue	Biomechanical Implications
Girard et al. (2011) [9]	Acute fatigue	Repeated sprint running	Instrumented track	High-intensity intermittent	Completion of 12 sprint bouts	Sprint performance, GRF	Vertical stiffness ↓; stride frequency ↓; leg stiffness unchanged
Girard et al. (2013) [7]	Acute fatigue	Continuous self-paced running (5 km)	Indoor track	Moderate–high intensity	Distance completion	Time, GRF	Vertical stiffness ↓; contact time ↑; stride length & frequency ↓
Koblbauer et al. (2014) [28]	Acute fatigue	Incremental treadmill running	Laboratory	Progressive to exhaustion	Volitional exhaustion	RPE, HR	Trunk flexion ↑; ankle eversion ↑
Fischer et al. (2015) [11]	Task-induced fatigue	CMJ fatigue before running	Laboratory	Short-duration maximal	Completion of jump protocol	Jump performance, GRF	COM displacement ↓; Kvert unchanged; step frequency ↑
Morin et al. (2015) [29]	Not fatigue-based	Sprint acceleration mechanics	Laboratory	Maximal sprint trials	Trial completion	GRF, EMG	Horizontal force linked to hamstring activation (no fatigue effects)
Giandolini et al. (2016) [13]	Extreme fatigue	Ultramarathon running (110 km)	Field	Prolonged endurance (>10 h)	Race completion	Distance, IMUs	Ankle ROM ↓; step frequency ↑; impact magnitude unchanged
Basile et al. (2017) [30]	Acute fatigue (pilot)	Continuous treadmill running	Laboratory	Submaximal prolonged	Time completion	Time	Highly individual GRF and strike-pattern responses
Wu et al. (2019) [5]	Task-classification study	Repeated sprint sessions + CMJ	Laboratory	Variable workloads	Protocol completion	Force–time PCA	Metabolic vs. neuromuscular fatigue signatures distinguished
Riazati et al. (2020) [31]	Acute fatigue	Energy-matched HIIT vs. MICR	Laboratory	Moderate–high intensity	Protocol completion	HR, strength loss	Hip ROM ↑; strength ↓; variability ↑ (greater after HIIT at individual level)
Yu et al. (2020) [32]	Acute fatigue	Run-to-exhaustion + CMJ	Laboratory	Submaximal continuous	Volitional exhaustion	Time	Joint loading ↑ despite unchanged jump height
Möhler et al. (2021) [8]	Acute fatigue	Middle-distance treadmill run	Instrumented treadmill	High intensity (~4 min)	Exhaustion	Time	Stance time ↑; vertical & leg stiffness ↓
Yu et al. (2021) [10]	Acute fatigue	Continuous treadmill running	Laboratory	Submaximal to exhaustion	Volitional exhaustion	Time	Hip, knee, ankle moments and powers ↑
Chalitsios et al. (2022) [33]	Acute fatigue	Exhaustive incremental treadmill run	Laboratory	Incremental maximal	Volitional exhaustion	RPE, ventilatory threshold	Trunk frontal/AP variability and GRF loading rate most fatigue-sensitive
Encarnación-Martínez et al. (2022) [14]	Differential fatigue	Central vs. peripheral protocols	Laboratory	Controlled workloads	Protocol completion	EMG, GRF	Central fatigue ↑ impact power; peripheral fatigue no effect
Gao et al. (2022) [17]	Acute fatigue	Prolonged treadmill running	Laboratory	Submaximal time-based	Time completion	Time	Hip and knee asymmetry ↑ post-fatigue
Möhler et al. (2022) [15]	Acute fatigue	Prolonged treadmill running	Laboratory	Moderate intensity	Exhaustion	Time	Motor variability ↑; CoM control ↓ but stability preserved
Munoz (2022) [12]	Acute fatigue	GXT + exhaustive running	Lab + overground	High intensity	VO_2_max + exhaustion	HR, time	Kvert & Kleg preserved via joint stiffness redistribution
Khaleghi Tazji et al. (2023) [16]	Acute fatigue	Multispeed treadmill running	Laboratory	Speed-dependent	Time-based	Time	Coordination ↓ and variability ↑, greater at higher speeds
Huang et al. (2023) [34]	Task-transfer fatigue	Squat fatigue + Y-Balance	Laboratory	Submaximal	Task completion	Balance scores	Joint ROM ↓; COP displacement ↑
Baggaley et al. (2024) [35]	Load-response (not fatigue)	Speed & grade perturbation running	Laboratory	Variable speeds & slopes	Protocol completion	Speed	Tibial strain ↑ with speed (fatigue–failure implication)
Jian et al. (2025) [36]	Acute fatigue (comparative)	Continuous treadmill running	Laboratory	Submaximal to exhaustion	Volitional exhaustion	Time	ACL stress ↑ in genu valgum group only
Kember et al. (2024) [37]	Task-induced fatigue	Repeated tuck jumps	Laboratory	Short-duration maximal	Task completion	Technique quality	Leg stiffness ↑; stabilization ↓ during landing
Mitschke et al. (2025) [38]	Real-world fatigue	Half-marathon race	Field	Prolonged endurance	Race completion	IMU metrics	Tibial acceleration ↑; contact time ↑
Einicke et al. (2018) [39]	Acute fatigue	Distance-induced running	Lab/field	Submaximal continuous	Distance completion	IMU dynamics	Wearables detected fatigue-related joint instability

**Table 5:** Characteristics of Fatigue Protocols and Methodological Patterns Across Included Studies.

Representative Studies (Refs)	Fatigue Protocol Characteristic	Observed Pattern Across Studies	Methodological and Biomechanical Implications
[7,8,10,11,13,15,16,17,18,31,33,38]	Fatigue type (acute vs. chronic)	Fatigue was operationalised as acute exposure, with biomechanics assessed immediately post-fatigue; no chronic or cumulative fatigue designs.	Findings reflect short-term neuromuscular and mechanical adaptations, not long-term injury mechanisms.
Continuous running: [7,8,10,28,31,33] Repeated sprints: [9] Endurance events: [13,38] Task-based: [5,32,37]	Primary fatigue modality	Continuous running dominated; fewer sprint-based, endurance race, or task-transfer protocols.	Different modalities stress distinct neuromuscular pathways, contributing to heterogeneous stiffness, coordination, and impact responses.
Laboratory: [8,10,11,15,31,33] Field: [7,9,13,38,39]	Running environment	Most studies were laboratory-based; fewer captured fatigue in real races or track conditions.	Labs improve measurement precision; field studies improve ecological validity but reduce control.
Short (<5 min): [5,11,37] Moderate (5–30 min): [7,8,10,31,33] Prolonged (>60 min): [13,38]	Fatigue duration	Exposure ranged from brief neuromuscular exhaustion to prolonged endurance accumulation.	Longer exposure associated with impact accumulation, coordination drift, and asymmetry, not only stiffness loss.
Submaximal: [31,33] Near-maximal: [7,8] Maximal/exhaustive: [9,11,14,28]	Fatigue intensity	Wide intensity range across studies.	Higher intensities produced faster reductions in force output and joint control, elevating injury-relevant loading.
Volitional exhaustion: [10,28,33] Fixed distance/time: [7,13,38] Protocol completion: [9,11,31]	Termination criteria	Termination criteria were heterogeneous and often non-physiological.	Limits comparability of true fatigue magnitude across studies.
RPE/time: [7,28,33] Force–time loss: [5,9,11] IMU metrics: [38,39]	Fatigue verification	Verification used subjective, mechanical, or wearable indicators, rarely standardised.	Non-uniform verification contributes to variability in biomechanical outcomes.
Single bout: [7,8,9,10,11,13,15,31,38]	Number of fatigue bouts	Nearly all studies used single-session fatigue exposure.	Prevents inference on residual or cumulative fatigue effects.
Immediate testing: [8,9,11,13,15,38]	Recovery allowance	Biomechanics typically recorded immediately post-fatigue.	Reflects peak fatigue state, not recovery-modulated adaptations.
CMJ/landing: [5,32,37] Balance: [34]	Transfer tasks	Several studies assessed fatigue transfer to jump or balance tasks.	Indicates fatigue effects extend to functional and screening tasks.
Individualised: [7,8,31] Fixed: [10,28,33]	Protocol standardisation	Some studies scaled workloads to individual capacity; others used fixed speeds.	Individualisation improves detection of fatigue-sensitive biomechanical responses.
Supervised lab protocols: [7,8,9,10,11,15,33]	Protocol supervision	Fatigue induction usually researcher-controlled.	Improves reliability of fatigue exposure and measurement.
Ethical safeguards: [13,30,36,38]	Safety controls	All studies applied ethical fatigue limits, especially in youth and endurance settings.	Ensures fatigue responses remain non-pathological.
High repeatability: [8,10,15] Lower repeatability: [13,38]	Repeatability	Treadmill protocols highly repeatable; race-based protocols variable.	Affects reproducibility of fatigue effects.
All studies	Intervention heterogeneity	Large heterogeneity in fatigue definition, modality, and verification.	Justifies narrative synthesis and limits meta-analytic pooling.

**Table 6:** Direction, Consistency, and Injury-Relevant Biomechanical Changes Under Fatigue.

Biomechanical Domain	Specific Variable	Direction and Nature of Change Under Fatigue	Key Supporting Studies (Refs)	Hypothesised Injury-Relevant Mechanical Pathway
Spatiotemporal	Ground contact time	Increased consistently	[7,8,10,13,15,28,33,38]	Greater cumulative tissue loading; reduced shock attenuation
	Step/stride length	Generally decreased	[7,8,10,38]	Higher step count → repetitive loading
	Cadence	Often unchanged or slightly reduced	[7,8,10,38]	Longer stance may increase joint load per step
Ankle kinematics	Peak dorsiflexion	Often increased	[10,28,31]	Increased Achilles and plantar fascia strain (theoretical)
	Ankle ROM	Variable; often slightly reduced	[11,28,31]	Reduced elastic recoil efficiency
Knee kinematics	Knee flexion (stance)	Increased during stance	[10,15,28,36]	Higher patellofemoral joint compression
	Frontal-plane motion	Variability and excursions increased	[17,33,36]	Elevated ACL and medial knee loading risk
Hip kinematics	Hip flexion	Increased proximal contribution	[10,36]	Greater lumbar–pelvic and hip extensor demand
Ankle kinetics	Push-off power	Reduced	[8,10,11,31]	Distal propulsion deficit → proximal compensation
	Functional stiffness	Often reduced (not universal)	[7,11,15]	Reduced energy storage and impact buffering
Knee kinetics	Knee moment	Often increased	[10,17,36]	Elevated quadriceps and patellar loading
	Knee work	Increased relative to ankle	[10,36]	Cumulative knee overuse risk
Hip kinetics	Hip power/work	Increased	[10,36]	Overload of hip extensors and trunk stabilisers
Impact loading	Vertical loading rate	Increased or poorly attenuated	[13,14,38]	Accelerated bone stress accumulation
	Tibial acceleration	Increased after prolonged fatigue and speed perturbation	[13,35,38]	Higher tibial bending stress → stress-fracture risk
Coordination & variability	Coordination variability	Increased (trunk–pelvis–hip)	[15,16,33]	Reduced movement stability and control
	Motor variability structure	Reorganised and less stable	[15,33]	Compromised neuromuscular regulation
Inter-limb asymmetry	Joint moment asymmetry	Increased	[17,38]	Unequal limb loading
	Stiffness asymmetry	Possible increase (limited evidence)	[12,17]	Uneven shock absorption
Neuromuscular	Force–time impulse	Reduced; altered force profiles	[5,11]	Impaired propulsion and load absorption
	Rate of force development	Reduced	[5]	Delayed stabilisation under dynamic load
Balance & stability	Postural control	Decreased dynamic balance	[34,37]	Reduced joint stabilisation capacity
	Landing stability	Impaired kinetic stabilisation	[37]	Increased non-contact injury susceptibility
Spring–mass behaviour	Vertical stiffness (Kvert)	Generally reduced	[7,9,11,12,13,15,38]	Reduced elastic energy storage and damping
	Leg stiffness (Kleg)	Mostly unchanged; ↓ in prolonged/field fatigue	[7,9,12,38]	Joint-level redistribution rather than global stiffness loss

## Data Availability

All evidence included in this systematic review is derived exclusively from previously published studies. No new data were generated or independently analyzed for the purposes of this research. Detailed information on the included studies, such as data extraction tables and methodological documentation, is available from the corresponding author upon reasonable request.
